# Early-Apoptotic Responses of Prostate and Melanoma Cancer Cells to 6 MV X-Ray Irradiation in the Presence of Ultrasmall Gold Nanoparticles

**DOI:** 10.3390/cimb48101071

**Published:** 2026-10-17

**Authors:** Farnaz Tabatabaie, Ava Bachari, Moshi Geso

**Affiliations:** 1 School of Sciences, RMIT University, Melbourne, VIC 3000, Australia; 2 The Pangenomics Lab, School of Science, RMIT University, Bundoora, VIC 3083, Australia; 3 School of Health & Biomedical Sciences, RMIT University, Bundoora, VIC 3083, Australia

**Keywords:** gold nanoparticles, ionizing radiation, apoptosis, Annexin V, radiation response, melanoma, prostate cancer, mitochondrial oxidative stress

## Abstract

Background: Gold nanoparticles (AuNPs) can modify cellular responses to ionizing radiation. We examined whether 1.9 nm AuNPs alter early apoptosis across a 0–8 Gy dose range in DU145 prostate and MM418-C1 melanoma cells. Methods: Cells were incubated with or without 0.5 mM AuNPs for 24 h, irradiated with 6 MV X-rays (0, 2, 4, 6, or 8 Gy), and analysed by Annexin V-FITC/PI flow cytometry 48 h later in three independent experiments. Only adherent cells were collected for analysis; cells detached into the culture supernatant were not included. A two-factor repeated-measures ANOVA tested radiation dose, AuNP exposure, and their interaction. Because sphericity could not be assessed reliably with *n* = 3, a conservative Geisser–Greenhouse lower-bound correction was also applied to effects involving dose. Results: The uncorrected analysis showed dose × AuNP interactions in DU145 [F(4,8) = 5.26, *p* = 0.0224, partial η^2^ = 0.725] and MM418-C1 [F(4,8) = 50.13, *p* = 1.05 × 10^−5^, partial η^2^ = 0.962]. After the conservative correction, the interaction remained significant in MM418-C1 (*p* = 0.0194) but not in DU145 (*p* = 0.149). In MM418-C1, AuNP exposure was associated with a higher early-apoptotic fraction at 2 Gy (+5.10 percentage points; Holm-adjusted *p* = 0.0019) but a lower fraction at 8 Gy (−17.03 percentage points; Holm-adjusted *p* = 0.0048). No DU145 dose-specific comparison remained significant after Holm correction. Conclusions: The AuNP-associated early-apoptotic response varied with radiation dose and cell line, most clearly in MM418-C1, while the DU145 interaction was less robust under the conservative correction. Given the small number of independent experiments and the 48 h endpoint, these findings should be interpreted as exploratory and not as a direct measure of total cytotoxicity or a specific cell-death mechanism.

## 1. Introduction

Ionizing radiation damages cells through direct energy deposition in critical biomolecules and through indirect effects initiated by water radiolysis and the generation of reactive oxygen species (ROS) [1,2,3,4]. Radiation-induced ROS can oxidize lipids, proteins, and nucleic acids and can perturb redox-sensitive signaling pathways that determine whether a cell undergoes repair, senescence, mitotic catastrophe, apoptosis, or other forms of death [5,6,7]. The relative contribution of these outcomes depends on radiation quality, dose, dose rate, cell type, and the time at which the biological response is measured.

**Figure 1:**
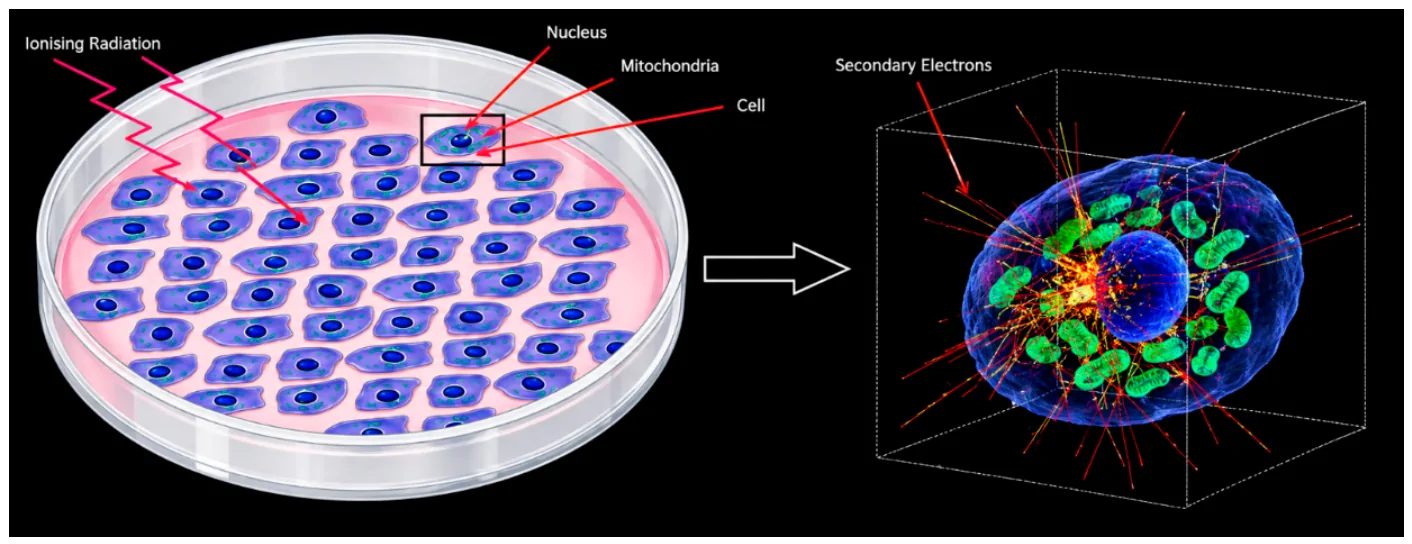
Schematic of a cultured cell monolayer exposed to ionizing radiation ((**left**); pink zigzag arrows) and an enlarged single cell (**right**) showing secondary-electron tracks (red and yellow lines) around the nucleus (dark blue) and mitochondria (green). Red arrows are labels pointing to the structures named. The diagram provides biological context; Adapted from Ref. [16] under the CC BY 4.0 license.

Mitochondria are both major regulators of cellular redox homeostasis and central participants in intrinsic apoptotic signaling [8,9,10,11,12,13]. Mitochondrial dysfunction can amplify ROS production and alter membrane potential, metabolic activity, and pro-apoptotic signaling (Figure 1). Consequently, mitochondrial ROS and mitochondrial signaling pathways have attracted increasing interest as potential targets for anticancer therapy [14,15]. In our previous study using DU145 and MM418-C1 cells, exposure to ionizing radiation in the presence of ultrasmall gold nanoparticles increased mitochondrial oxidative damage in a radiation dose-dependent manner [16]. This observation provided a rationale for examining whether the same treatment conditions also modify the early apoptotic fraction of the irradiated cell population.

Gold nanoparticles have been investigated as radiation-response modifiers because their high atomic number can increase local secondary-electron production and because their physicochemical properties can alter oxidative stress and cellular signaling [17,18,19,20,21,22]. Importantly, the magnitude and mechanism of radiosensitization depend on nanoparticle size, coating, concentration, intracellular distribution, radiation energy, and biological context. At megavoltage photon energies, biological mechanisms may contribute substantially to observed radiosensitization, so an increase in apoptosis cannot be assumed to arise from physical dose enhancement alone.

Mechanistically, AuNP-mediated radiosensitization is best viewed as a combination of physical, chemical, and biological effects rather than as a simple increase in macroscopic absorbed dose. At megavoltage energies, the predicted bulk dose enhancement from gold is modest, yet nanoscale secondary-electron emission and radiation-induced radical chemistry can amplify local oxidative stress. Downstream effects reported for AuNPs include altered redox homeostasis, mitochondrial dysfunction, impaired DNA-repair signaling, cell-cycle perturbation, and activation of apoptotic pathways [23,24]. Recent work has also shown that ROS production and clonogenic radiosensitization depend strongly on AuNP size and surface coating, reinforcing the need to interpret biological response in the context of nanoparticle physicochemical properties [25,26].

The novelty of the present work is therefore not the use of ultrasmall AuNPs alone. Rather, it is the analysis of the early-apoptotic response across the 0–8 Gy radiation dose range after clinically relevant 6 MV irradiation in two distinct human cancer cell models, considered alongside mitochondrial oxidative-stress and clonogenic findings already published from the same broader experimental programme. The Annexin V/PI endpoint reported here was not included in our earlier peer-reviewed publication [16]. The factorial design also allows direct testing of whether the association between AuNP exposure and early apoptosis changes with radiation dose. Because only three independent experiments were performed, the inferential findings are interpreted as exploratory.

Annexin V/propidium iodide (PI) flow cytometry provides a practical method for resolving viable, early-apoptotic, and membrane-compromised cell populations [27,28,29,30]. Annexin V binding identifies externalization of phosphatidylserine, an early feature of apoptosis, whereas PI uptake indicates loss of membrane integrity. Although this assay quantifies apoptotic state, it does not by itself identify a mitochondrial mechanism. Accordingly, the present study was designed to quantify early apoptosis after 0–8 Gy irradiation, with and without 1.9 nm AuNPs, in DU145 prostate carcinoma and MM418-C1 melanoma cells, and to interpret the dose response in the context of our previously demonstrated mitochondrial oxidative stress [16].

## 2. Materials and Methods

### 2.1. Cell Culture

Two human cancer cell lines were studied: MM418-C1 melanoma cells and DU145 prostate carcinoma cells. MM418-C1 cells were supplied by Prof. Nicholas Hayward and Peter Parsons (Queensland Institute of Medical Research, Brisbane, Australia) and were maintained in RPMI 1640 (Gibco) supplemented with 10% fetal bovine serum (FBS) and 1% penicillin-streptomycin. DU145 cells were obtained from ATCC (Manassas, VA, USA) and maintained in MEM Alpha + GlutaMAX supplemented with 15 mM HEPES, 10% FBS, and 1% penicillin-streptomycin. Cells were cultured at 37 °C in a humidified 5% CO_2_ atmosphere.

Cells were grown in 75 cm^2^ culture flasks to approximately 80% confluence and sub-cultured at a 1:3 ratio using trypsin-EDTA (Gibco). For experiments, cells were seeded in 6-well plates at 2 × 10^6^ cells/mL in 1 mL of complete culture medium per well.

### 2.2. Gold Nanoparticle Preparation and Exposure

A biocompatible preparation of spherical, water-soluble, organic-skeleton-stabilized AuNPs with a supplier-reported 1.9 nm gold-core diameter was obtained from Nanoprobes Inc. (Yaphank, NY, USA) [31]. The stock preparation was diluted in complete culture medium to a final AuNP concentration of 0.5 mM (0.0985 mg/mL), as described in our earlier work [16]. The 0.5 mM concentration refers to the molar concentration of elemental gold and corresponds to 0.0985 mg/mL of gold in the working preparation. Control cultures received the corresponding complete medium without AuNPs. Both cell lines were incubated with AuNPs for 24 h before irradiation. The same nanoparticle concentration and incubation period were used for all AuNP-treated groups.

These nanoparticles were purchased as a finished commercial preparation; they were not synthesized or purified by the authors. Accordingly, no study-specific STEM/TEM, XRD, EDS, UV-Vis, DLS, or zeta-potential measurements were performed on the historical experimental lot. The stated 1.9 nm core size and organic stabilization are based on supplier information and the cited source literature.

Independent published work has characterized a commercial 1.9 nm AuroVist™ formulation from the same supplier by transmission electron microscopy, reporting a monodispersed population with a median particle diameter of approximately 2.0–2.5 nm; that study also examined uptake and intracellular localization in DU145 cells [32]. These published data provide useful context for the supplier’s 1.9 nm formulation but are not presented as batch-specific characterization of the material used in the present experiments.

### 2.3. Irradiation

**Figure 2:**
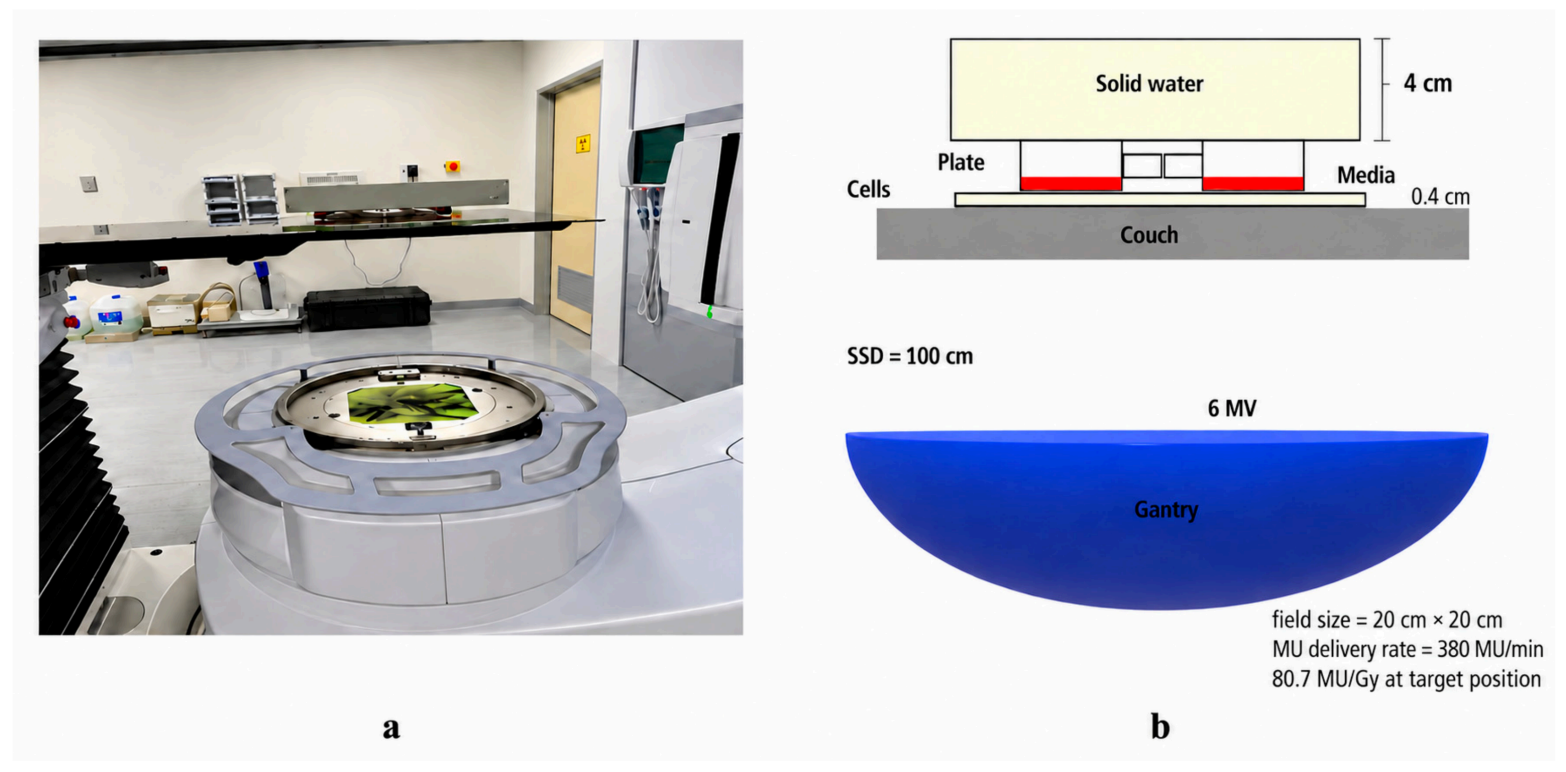
Cell-irradiation arrangement at the ARPANSA linear accelerator. (**a**) Photograph of the experimental setup. (**b**) Schematic of the plate, water-equivalent buildup/backscatter arrangement, and 6 MV irradiation geometry.

Cells were irradiated using 6 MV X-rays from an Elekta Synergy medical linear accelerator (Elekta AB, Stockholm, Sweden) at the Australian Radiation Protection and Nuclear Safety Agency (ARPANSA), Yallambie, Victoria, Australia (Figure 2). Single-fraction doses of 0, 2, 4, 6, and 8 Gy were delivered to cultures with and without AuNPs. Irradiations used a source-to-surface distance (SSD) of 100 cm and a 20 × 20 cm^2^ field. The LINAC was operated at a monitor-unit delivery rate of 380 MU/min. For this irradiation geometry, the calibration at the target position was 80.7 MU/Gy, corresponding to an absorbed-dose rate of approximately 4.71 Gy/min. The beam was delivered from below, through the couch, to plates resting on a 0.4 cm water-equivalent slab (Figure 2b). Because the cells formed an adherent monolayer at the bottom of the wells, nearest the beam entrance, a 4 cm water-equivalent layer was placed on top of the plates to provide backscatter, with a thin layer of culture medium above the cellular layer. Irradiation geometry and buildup/backscatter conditions were kept consistent across treatment groups, and output was checked with an ionization chamber before biological irradiation.

Before the biological irradiations, the irradiation geometry was established under the same buildup and backscatter conditions used for the cell plates, and output was checked with an ionization chamber. The prescribed dose was then delivered using the clinically calibrated LINAC monitor-unit system. This pre-irradiation dosimetric verification was used to confirm delivery of the intended 0–8 Gy dose series; nanoparticle-scale dose enhancement was not measured directly and is not inferred from these chamber measurements.

### 2.4. Annexin V-FITC/PI Flow-Cytometry Assay

Apoptotic state was assessed using Annexin V-FITC/PI staining followed by flow cytometry [30,33,34,35]. At 48 h after irradiation, adherent cells were harvested and prepared for Annexin V-FITC/PI analysis. Floating cells in the culture supernatant were not collected. Aliquots of 200 µL containing approximately 2 × 10^5^ cells were stained with 5 µL Annexin V-FITC (Pharmingen, San Diego, CA, USA) and 20 µL binding buffer. Samples were gently mixed and incubated for 15 min at room temperature in the dark. Immediately before acquisition, 2 µL PI (1 mg/mL; Cedarlane Laboratories, Hornby, ON, Canada) was added. Samples were analysed without washing using a BD LSRFortessa flow cytometer (BD Biosciences, San Jose, CA, USA) equipped with blue (488 nm) and yellow-green (561 nm) lasers. At least 10,000 events within the final gated cell population were analysed for each sample.

**Figure 3:**
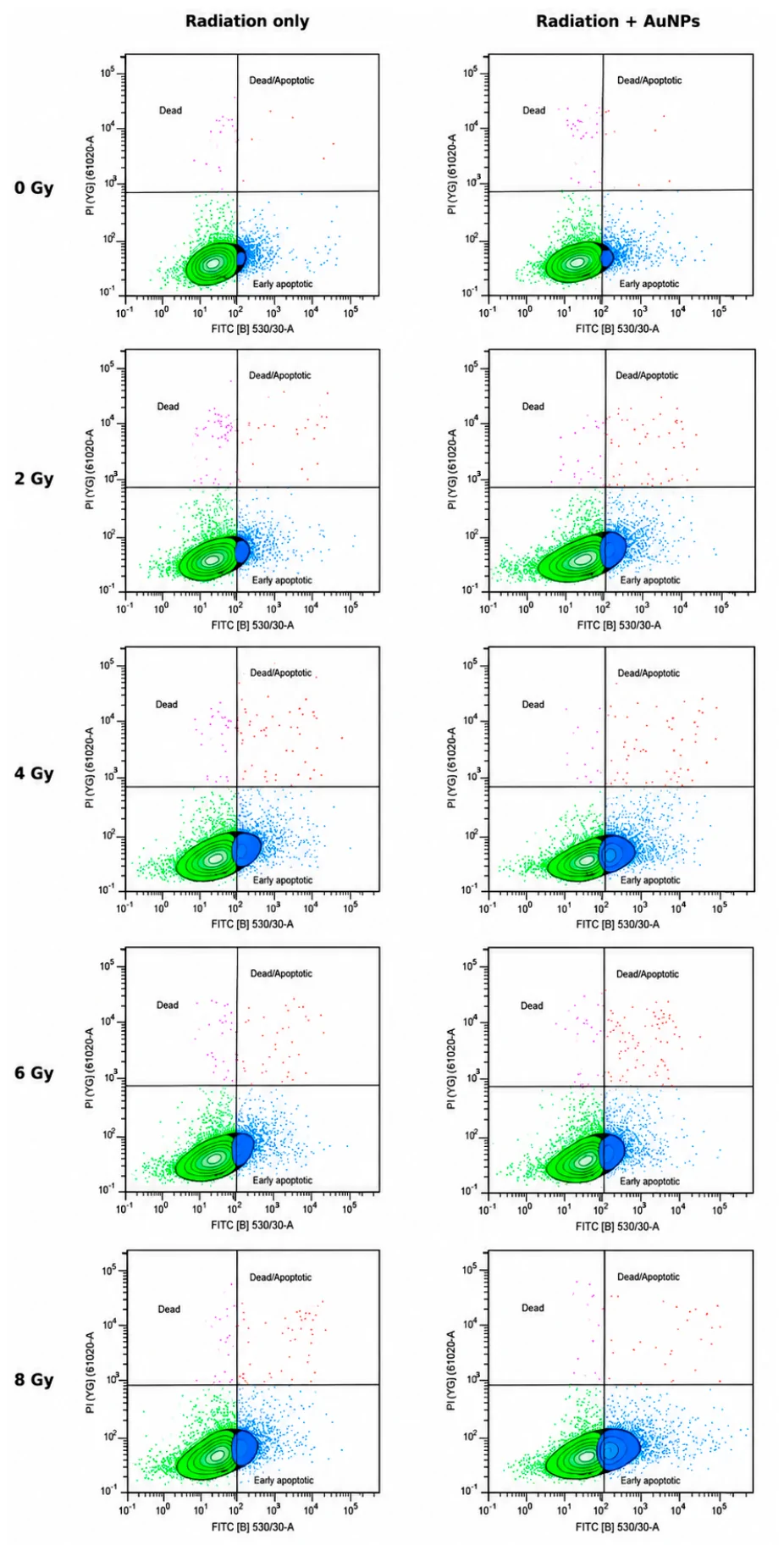
Representative Annexin V-FITC/PI contour plots for DU145 cells exposed to 0, 2, 4, 6, or 8 Gy in the absence or presence of AuNPs. Annexin V-FITC is shown on the *x*-axis and PI on the *y*-axis. The lower-right quadrant represents early apoptotic cells (Annexin V-positive/PI-negative). Event colours correspond to the quadrant populations: viable (Annexin V-negative/PI-negative), early apoptotic (Annexin V-positive/PI-negative), Annexin V-positive/PI-positive (dead/apoptotic), and Annexin V-negative/PI-positive (dead).

A sequential gating strategy was applied before Annexin V/PI quadrant analysis. An FSC-A versus SSC-A gate was first used to exclude cellular debris and low-scatter events, followed by an FSC-A versus FSC-H diagonal gate for singlet discrimination. Annexin V-FITC versus PI populations were then analysed using a single global quadrant template that was applied consistently across experimental runs to maintain uniform quadrant placement. Annexin V-FITC was detected through a 530/30 nm bandpass filter using 488 nm excitation, while PI was detected through a 610/20 nm bandpass filter using 561 nm excitation (detector channels FITC [B] 530/30-A and PI [YG] 610/20-A, as labelled in Figure 3). Quadrant boundaries were set using an unstained control sample to establish the baseline of cellular autofluorescence, with the lines positioned so that approximately 99% of healthy, untreated cells fell within the lower-left (double-negative) quadrant. The quadrant grid was then fixed and applied uniformly to all treatment groups. Single-stained controls and fluorescence-compensation settings could not be confirmed from the archived acquisition records; whether compensation was applied is therefore not reported, and residual spectral spillover between the FITC and PI channels cannot be excluded. Annexin V-negative/PI-negative cells were classified as viable, while Annexin V-positive/PI-negative cells were classified as early apoptotic and constituted the primary endpoint analysed in this study. Annexin V-positive/PI-positive cells were interpreted as late apoptotic or secondary necrotic, whereas Annexin V-negative/PI-positive cells were classified as nonviable/PI-positive.

### 2.5. Experimental Design and Statistical Analysis

**Figure 4:**
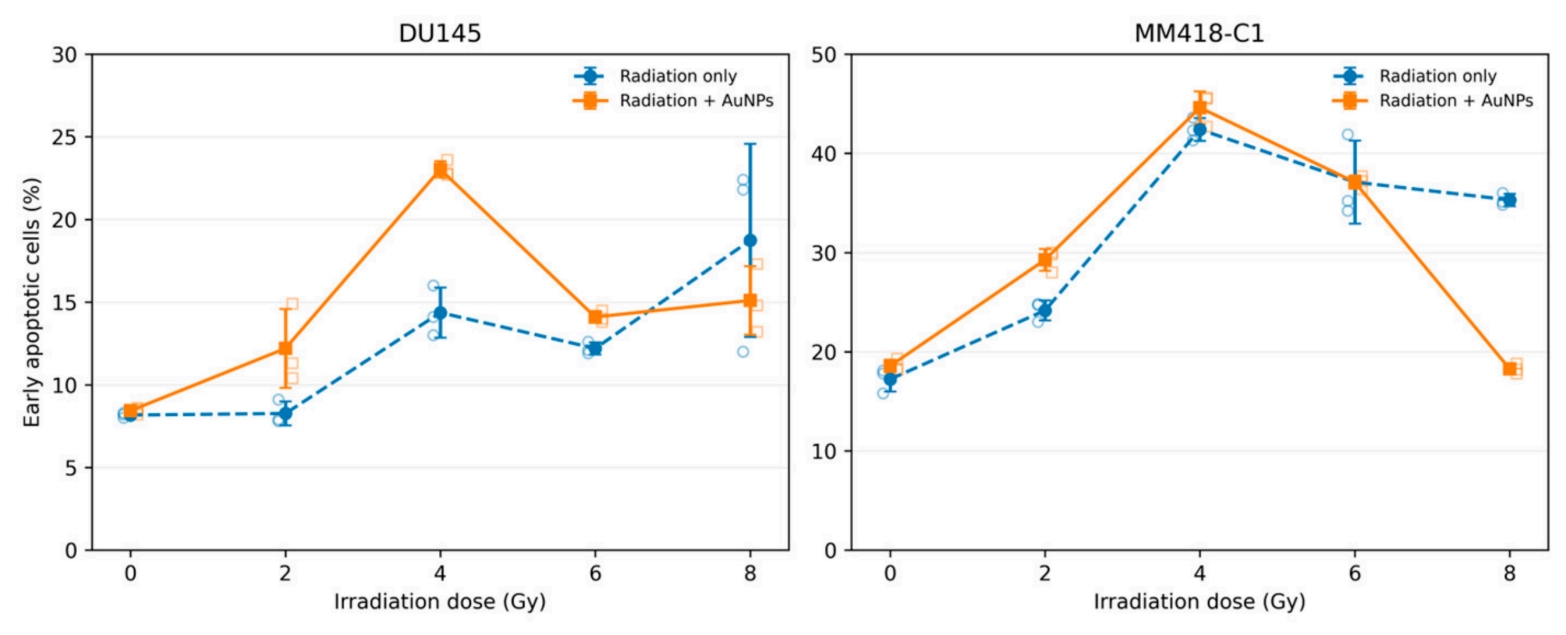
Early-apoptotic fraction in DU145 and MM418-C1 cells following irradiation at 0–8 Gy in the absence or presence of AuNPs. Open symbols show the three individual experiment-level values for each condition; filled symbols and error bars show mean ± SD (*n* = 3). Straight lines connect successive group means as visual guides only; no continuous dose–response model was fitted.

Each cell line was evaluated at five radiation doses (0, 2, 4, 6, and 8 Gy) in the absence and presence of 0.5 mM AuNPs. The experimental design and primary readout are summarized in Table 1. Three independent biological experiments were performed, with each experiment containing the complete dose × AuNP condition set. Independent experiments were performed on separate days using cells from separate passages. The same sequential gating approach and global Annexin V/PI quadrant template were applied across experimental runs. The Annexin V-positive/PI-negative percentage from each experiment was analysed as exported, without post hoc renormalization of the quadrant values. Data are presented as mean ± standard deviation (SD), and individual experiment-level values are shown in Figure 4. For each cell line, a two-factor repeated-measures ANOVA was performed with radiation dose and AuNP exposure as within-experiment factors and experimental run as the subject. Effect size is reported as partial eta squared (partial η^2^). Because sphericity cannot be tested reliably with *n* = 3 and five dose levels, effects involving radiation dose were additionally evaluated with the conservative Geisser–Greenhouse lower-bound correction (ε = 1/4; df = 1, 2), and both uncorrected and lower-bound *p*-values are reported. The AuNP main effect has one degree of freedom and requires no correction. Where the dose × AuNP interaction was significant, paired comparisons between AuNP-treated and untreated samples were performed at each radiation dose using two-sided paired *t*-tests. Ninety-five percent confidence intervals (CIs) were calculated for the paired mean differences, and Holm correction was applied across the five dose-specific comparisons within each cell line. Exact *p*-values are reported. Given the small number of independent experiments (*n* = 3), all inferential results are interpreted cautiously as exploratory. Statistical analyses were performed in Python 3.13.5 using statsmodels 0.14.6 and SciPy 1.17.0.

### 2.6. Use of Generative Artificial Intelligence

During preparation of this manuscript, the authors used OpenAI ChatGPT (GPT-5.6 Sol) for assistance with manuscript writing, language editing, and checking the statistical-analysis code and presentation. Statistical calculations were performed using standard Python 3.13.5 packages (statsmodels and SciPy) and were reviewed by the authors. Generative AI was not used for experimental design, data collection, or generation or alteration of experimental results. The authors reviewed and edited all output and take full responsibility for the content of the publication.

## 3. Results

### 3.1. Representative Annexin V/PI Profiles

Representative DU145 flow-cytometry contour plots across the 0–8 Gy dose range are shown in Figure 3 and are provided as illustrative profiles only. Quantitative comparisons focus on the Annexin V-positive/PI-negative early-apoptotic fraction.

### 3.2. Early-Apoptotic Fraction Across Irradiation Doses

Individual experiment-level values and group means are shown in Figure 4. In DU145 cells, the AuNP-treated mean was higher than the radiation-only mean at 2, 4, and 6 Gy, with the largest positive mean separation at 4 Gy; at 8 Gy, the direction of the mean difference reversed. In MM418-C1 cells, the AuNP-treated mean was higher at 2 and 4 Gy, was identical to the radiation-only mean at 6 Gy, and was markedly lower at 8 Gy. These patterns indicate that the association between AuNP exposure and the early-apoptotic fraction was not uniform across radiation dose. In both cell lines the mean early-apoptotic fraction was lower at 6 Gy than at 4 Gy in both treatment arms; between 6 and 8 Gy, the only pronounced further decline occurred in AuNP-treated MM418-C1 cells, whereas radiation-only MM418-C1 cells remained at a similar level and radiation-only DU145 cells showed a higher mean at 8 Gy than at 6 Gy (Figure 4).

Two-factor repeated-measures ANOVA demonstrated a significant effect of radiation dose in DU145 cells [F(4,8) = 33.34, *p* = 0.000049, partial η^2^ = 0.943] and MM418-C1 cells [F(4,8) = 217.33, *p* = 3.43 × 10^−8^, partial η^2^ = 0.991]. The overall main effect of AuNP exposure was not statistically significant in DU145 [F(1,2) = 4.00, *p* = 0.1837, partial η^2^ = 0.666] or MM418-C1 [F(1,2) = 11.43, *p* = 0.0775, partial η^2^ = 0.851]. Importantly, the dose × AuNP interaction was significant in both DU145 [F(4,8) = 5.26, *p* = 0.0224, partial η^2^ = 0.725] and MM418-C1 [F(4,8) = 50.13, *p* = 1.05 × 10^−5^, partial η^2^ = 0.962], indicating that the AuNP-associated difference depended on radiation dose (Table 2). Under the conservative Geisser–Greenhouse lower-bound correction (df = 1, 2), the dose effect remained significant in DU145 (*p* = 0.0287) and MM418-C1 (*p* = 0.0046), and the dose × AuNP interaction remained significant in MM418-C1 (*p* = 0.0194) but not in DU145 (*p* = 0.149); the DU145 interaction should therefore be regarded as suggestive.

Dose-specific paired comparisons were used to examine the interaction (Table 3). In DU145 cells, the largest positive mean difference occurred at 4 Gy (+8.67 percentage points; 95% CI 4.64 to 12.69; unadjusted *p* = 0.0114), but no individual dose-specific comparison remained significant after Holm correction (adjusted *p* = 0.0572 at 4 Gy). In MM418-C1 cells, AuNP exposure was associated with a higher early-apoptotic fraction at 2 Gy (+5.10 percentage points; 95% CI 4.67 to 5.53; Holm-adjusted *p* = 0.0019). At 8 Gy, the direction reversed, with the AuNP-treated group showing a lower early-apoptotic fraction than radiation alone (−17.03 percentage points; 95% CI −19.57 to −14.50; Holm-adjusted *p* = 0.0048). The 4 Gy and 6 Gy MM418-C1 contrasts were not significant after correction.

### 3.3. Factorial and Dose-Specific Statistical Analyses

The factorial ANOVA results are summarized in Table 2. Dose-specific paired contrasts, including 95% confidence intervals and Holm-adjusted *p*-values, are summarized in Table 3. Individual experiment-level values are shown in Figure 4. The individual values plotted in Figure 4 are provided in Supplementary Table S1, with experimental-run identifiers showing which paired measurements originated from the same independent experiment.

## 4. Discussion

The present study examines how AuNP exposure modifies the early-apoptotic response to 6 MV irradiation at a defined 48 h endpoint. The main statistical finding is a significant dose × AuNP interaction in both DU145 and MM418-C1 cells (although the DU145 interaction was not robust to a conservative sphericity correction), whereas the overall main effect of AuNP exposure was not statistically significant when averaged across all radiation doses. The effect associated with AuNP exposure was therefore dose-dependent rather than uniformly positive or negative across the tested range.

In DU145 cells, AuNP-treated groups showed higher mean early-apoptotic fractions at 2, 4, and 6 Gy, with the largest positive difference at 4 Gy (+8.67 percentage points). However, none of the individual DU145 dose comparisons remained significant after Holm correction, and the mean difference reversed at 8 Gy. In MM418-C1 cells, the pattern was different: the AuNP-associated increase at 2 Gy remained significant after correction, the difference at 4 Gy was smaller and not significant, there was no mean difference at 6 Gy, and the direction reversed strongly at 8 Gy. This cell-line- and dose-specific behaviour is more informative than describing AuNP exposure as producing a single uniform radiosensitizing effect on early apoptosis.

The lower early-apoptotic fraction observed with AuNPs at 8 Gy, particularly in MM418-C1 cells, should not be interpreted as evidence that AuNPs are protective or that total radiation injury is reduced. Annexin V-FITC/PI provides a snapshot of cell state at one time point. Cells may progress through early apoptosis at different rates, move into late apoptosis or secondary necrosis, undergo other forms of cell death, or be absent from the analysed recoverable population by 48 h. Because the other Annexin V/PI populations are not analysed in this study, the biological basis of the high-dose reversal cannot be determined from this endpoint alone. The decline from 4 to 6 Gy was seen in both treatment arms of both cell lines, whereas the additional decline at 8 Gy was confined to AuNP-treated MM418-C1 cells.

The non-linear dose patterns should also not be equated with classical low-dose hyper-radiosensitivity (HRS). HRS is primarily defined using clonogenic survival at doses generally below approximately 0.5 Gy [2], whereas the present study evaluates an Annexin V-positive/PI-negative state after doses of 0–8 Gy. A similar turning point around 4 Gy was observed in our previously published mitochondrial ROS and bioenergetic measurements [16], which provides useful biological context but does not establish the mechanism responsible for the present dose × AuNP interaction.

AuNP-mediated radiation response is biologically complex and may involve local secondary-electron production, ROS generation, organelle stress, altered redox signalling, and effects on DNA repair and cell-cycle regulation [17,20,21,36]. Our previous work in the same broader experimental programme showed increased mitochondrial superoxide and altered mitochondrial respiratory stress after combined AuNP and irradiation exposure [16]. Clonogenic assays from that programme also demonstrated reduced long-term colony-forming survival, with reported DEF values of 1.17 for DU145 and 1.21 for MM418-C1 [16]. These findings support an AuNP-associated radiosensitizing effect in the broader experimental system, but the present Annexin V/PI analysis should be interpreted specifically as a 48 h early-apoptosis endpoint rather than as an independent measure of clonogenic radiosensitization or a mitochondria-specific pathway.

The differences between DU145 and MM418-C1 are also notable. The two cell lines differed not only in the magnitude of the response but also in how the direction of the AuNP-associated difference changed with dose. Possible contributors include differences in nanoparticle uptake, basal redox state, antioxidant capacity, cell-cycle distribution, apoptotic competence, or intrinsic radiation response. These mechanisms were not measured directly in the present apoptosis experiment, so they remain plausible explanations rather than established causes. MM418-C1 was included as a contrasting melanoma model and should not be considered representative of all melanomas.

Several limitations should be considered when interpreting these findings. The study was performed in vitro using two cancer cell lines and a single AuNP size and concentration. No normal-cell comparator was included, so tumour selectivity cannot be concluded from these data. In addition, the commercial AuNP preparation was not independently characterized for hydrodynamic size, surface charge, or aggregation state, and cellular uptake was not quantified. Compensation status could not be verified from the archived records, so spectral spillover between detection channels cannot be excluded. These factors may influence biological response and should be addressed in future studies.

Timing remains an important consideration. All samples were assessed 48 h after irradiation, and early apoptosis is a dynamic state. The dose-specific interaction identified here therefore describes the distribution of cells in the early-apoptotic compartment at this particular time point; it should not be extrapolated to the complete temporal course of radiation-induced cell death. Multiple post-irradiation measurements would be needed to determine whether the high-dose reversals reflect altered kinetics, redistribution to other death states, or another biological process.

The statistical analysis accounts for the two-factor design and the matched structure of the three independent experiments. Nevertheless, *n* = 3 is small, which limits precision and makes repeated-measures model assumptions difficult to assess robustly. In particular, sphericity cannot be tested with three subjects; the uncorrected F-tests assume it, and the conservative Geisser–Greenhouse lower-bound correction removed the significance of the DU145 interaction. The exact *p*-values, confidence intervals, and effect sizes should therefore be interpreted as exploratory rather than as definitive estimates of the magnitude of the interaction. The significant interaction terms provide evidence that the early-apoptotic response associated with AuNP exposure changes with radiation dose, but they do not by themselves establish a mechanism or clinically meaningful radiosensitization.

Taken together, the results show that the AuNP-associated early-apoptotic response is dose- and cell-line-dependent and is not uniformly increased across the radiation range. Future work should include larger numbers of independent experiments, normal-cell comparators, detailed nanoparticle characterization and uptake measurements, multiple post-irradiation time points, and complementary endpoints such as clonogenic survival, caspase activation, mitochondrial membrane potential, cytochrome-c release, and DNA-damage markers within the same experimental series.

## 5. Conclusions

Under the conditions studied, the association between AuNP exposure and the Annexin V-positive/PI-negative early-apoptotic fraction depended on radiation dose and cell line. A dose × AuNP interaction was identified in MM418-C1 cells and, less robustly, in DU145 cells (where it did not survive a conservative sphericity correction), indicating that the effect of AuNP exposure on early apoptosis was not uniform across the radiation dose range. The response pattern also differed between the two cell lines, particularly at higher doses. Only adherent cells were collected for analysis; therefore, the reported percentages do not include cells that had detached into the culture supernatant following treatment. Given the small number of independent experiments and the time-specific nature of Annexin V/PI analysis, these findings should be interpreted as evidence of a dose-dependent interaction in the early-apoptotic response rather than as a direct measure of total cytotoxicity, clonogenic radiosensitization, or a specific mitochondrial mechanism.

## Figures and Tables

**Table 1:** Experimental design and primary readout.

Parameter	Condition
Cell lines	DU145 prostate carcinoma; MM418-C1 melanoma
AuNP exposure	0 or 0.5 mM; 1.9 nm core diameter; 24 h pre-incubation
Radiation	6 MV X-rays; 0, 2, 4, 6, or 8 Gy; single fraction
Primary endpoint	Early apoptotic fraction: Annexin V-FITC positive/PI negative
Flow-cytometry acquisition	≥10,000 gated singlet events per sample; common Annexin V/PI quadrant template across runs
Replicates	Three independent experiments

**Table 2:** Two-factor repeated-measures ANOVA for the early-apoptotic fraction.

Cell Line	Effect	F (df1, df2)	Exact *p*	Partial η^2^
DU145	Radiation dose	33.34 (4, 8)	0.000049	0.943
DU145	AuNP exposure	4.00 (1, 2)	0.183650	0.666
DU145	Dose × AuNP	5.26 (4, 8)	0.022435	0.725
MM418-C1	Radiation dose	217.33 (4, 8)	3.43 × 10^−8^	0.991
MM418-C1	AuNP exposure	11.43 (1, 2)	0.077461	0.851
MM418-C1	Dose × AuNP	50.13 (4, 8)	1.05 × 10^−5^	0.962

Note to Table 2: Conservative Geisser–Greenhouse lower-bound *p*-values (df = 1, 2): DU145, radiation dose 0.0287 and dose × AuNP 0.149; MM418-C1, radiation dose 0.0046 and dose × AuNP 0.0194. The AuNP main effect has df = 1 and is not corrected.

**Table 3:** Dose-specific paired comparisons of early apoptosis (+AuNP minus radiation-only).

Cell Line	Dose (Gy)	Mean Difference (pp)	95% CI	Exact *p*	Holm-adjusted *p*
DU145	0	+0.27	0.12 to 0.41	0.0153	0.0611
DU145	2	+3.93	−0.20 to 8.07	0.0549	0.1098
DU145	4	+8.67	4.64 to 12.69	0.0114	0.0572
DU145	6	+1.90	0.66 to 3.14	0.0223	0.0669
DU145	8	−3.63	−22.89 to 15.63	0.5022	0.5022
MM418-C1	0	+1.33	−3.34 to 6.01	0.3447	0.6894
MM418-C1	2	+5.10	4.67 to 5.53	0.000384	0.0019
MM418-C1	4	+2.20	−0.25 to 4.65	0.0608	0.1823
MM418-C1	6	0.00	−10.12 to 10.12	1.0000	1.0000
MM418-C1	8	−17.03	−19.57 to −14.50	0.00120	0.0048

Abbreviations: pp, percentage points; CI, confidence interval. Holm-adjusted *p*-values correct for the five dose-specific comparisons within each cell line.

## Data Availability

The raw data supporting the conclusions of this article will be made available by the authors on request.

## Supplementary Materials

The following supporting information can be downloaded at https://www.mdpi.com/article/10.3390/cimb48101071/s1.

## References

[1] Lehnert B.E., Iyer R. (2002). Exposure to low-level chemicals and ionizing radiation: Reactive oxygen species and cellular pathways. Hum. Exp. Toxicol..

[2] Calabrese E.J. (2017). Biological Effects of Low Level Exposures to Chemical and Radiation.

[3] Sies H., Belousov V.V., Chandel N.S., Davies M.J., Jones D.P., Mann G.E., Murphy M.P., Yamamoto M., Winterbourn C. (2022). Defining roles of specific reactive oxygen species (ROS) in cell biology and physiology. Nat. Rev. Mol. Cell Biol..

[4] Azzam E.I., Jay-Gerin J.-P., Pain D. (2012). Ionizing radiation-induced metabolic oxidative stress and prolonged cell injury. Cancer Lett..

[5] Hancock J.T., Desikan R., Neill S.J. (2001). Role of reactive oxygen species in cell signalling pathways. Biochem. Soc. Trans..

[6] Pfeffer C.M., Singh A.T.K. (2018). Apoptosis: A target for anticancer therapy. Int. J. Mol. Sci..

[7] Eriksson D., Stigbrand T. (2010). Radiation-induced cell death mechanisms. Tumor Biol..

[8] Forman H.J., Kennedy J.A. (1974). Role of superoxide radical in mitochondrial dehydrogenase reactions. Biochem. Biophys. Res. Commun..

[9] Kowaltowski A.J., de Souza-Pinto N.C., Castilho R.F., Vercesi A.E. (2009). Mitochondria and reactive oxygen species. Free Radic. Biol. Med..

[10] Hernansanz-Agustín P., Enríquez J.A. (2021). Generation of reactive oxygen species by mitochondria. Antioxidants.

[11] Zhao Y., Lv B., Xue G., Sun Y., Cao J. (2023). Smart nanosystem-mediated inhibition of mitochondrial respiration for enhanced phototherapy-induced antitumor immunity. Int. J. Nanomed..

[12] Lee Y.G., Park D.H., Chae Y.C. (2022). Role of mitochondrial stress response in cancer progression. Cells.

[13] Ma C., Xia F., Kelley S.O. (2020). Mitochondrial targeting of probes and therapeutics to the powerhouse of the cell. Bioconjug. Chem..

[14] Yang Y., Karakhanova S., Hartwig W., D’Haese J.G., Philippov P.P., Werner J., Bazhin A.V. (2016). Mitochondria and mitochondrial ROS in cancer: Novel targets for anticancer therapy. J. Cell. Physiol..

[15] Kafkova A., Trnka J. (2020). Mitochondria-targeted compounds in the treatment of cancer. Neoplasma.

[16] Tabatabaie F., Franich R., Feltis B., Geso M. (2022). Oxidative damage to mitochondria enhanced by ionizing radiation and gold nanoparticles in cancer cells. Int. J. Mol. Sci..

[17] Laprise-Pelletier M., Simão T., Fortin M.A. (2018). Gold nanoparticles in radiotherapy and recent progress in nanobrachytherapy. Adv. Healthc. Mater..

[18] Shahhoseini E., Nakayama M., Piva T.J., Geso M. (2021). Differential effects of gold nanoparticles and ionizing radiation on cell motility between primary human colonic and melanocytic cells and their cancerous counterparts. Int. J. Mol. Sci..

[19] Borran A.A., Aghanejad A., Farajollahi A., Barar J., Omidi Y. (2018). Gold nanoparticles for radiosensitizing and imaging of cancer cells. Radiat. Phys. Chem..

[20] Vlastou E., Pantelis E., Efstathopoulos E.P., Karaiskos P., Kouloulias V., Platoni K. (2022). Quantification of nanoscale dose enhancement in gold nanoparticle-aided external photon beam radiotherapy. Cancers.

[21] Kouri M.A., Spyratou E., Kalkou M.-E., Patatoukas G., Angelopoulou E., Tremi I., Havaki S., Gorgoulis V.G., Kouloulias V., Platoni K. (2023). Nanoparticle-mediated radiotherapy: Unraveling dose enhancement and apoptotic responses in cancer and normal cell lines. Biomolecules.

[22] Boisselier E., Astruc D. (2009). Gold nanoparticles in nanomedicine: Preparations, imaging, diagnostics, therapies and toxicity. Chem. Soc. Rev..

[23] Bemidinezhad A., Mirzavi F., Gholamhosseinian H., Gheybi F., Soukhtanloo M. (2023). Gold-containing liposomes and glucose-coated gold nanoparticles enhance the radiosensitivity of B16F0 melanoma cells via increasing apoptosis and ROS production. Life Sci..

[24] Kouri M.A., Georgopoulos A., Manios G.E., Maratou E., Spathis A., Chatziioannou S., Platoni K., Efstathopoulos E.P. (2024). Preliminary Study on Lutetium-177 and Gold Nanoparticles: Apoptosis and Radiation Enhancement in Hepatic Cancer Cell Line. Curr. Issues Mol. Biol..

[25] Loscertales E., López-Méndez R., Mateo J., Fraile L.M., Udias J.M., Espinosa A., España S. (2025). Impact of gold nanoparticle size and coating on radiosensitization and generation of reactive oxygen species in cancer therapy. Nanoscale Adv..

[26] Biswal P., Vijay G.V., Krouse G., Mai P.M.Q., Lakshmisha B.M., Shahbaz S.K., Taba M.Y., Sabbagh A., Cheung L.W.T., Mackeyev Y. (2026). Tumor radiosensitization with gold nanoparticles: Evolving strategies to improve tumoral gold uptake and catalyze future clinical translation. ACS Nano Med..

[27] Boreham D.R., Gale K.L., Maves S.R., Walker J.A., Morrison D.P. (1996). Radiation-induced apoptosis in human lymphocytes: Potential as a biological dosimeter. Health Phys..

[28] Collins R.J., Harmon B.V., Gobé G.C., Kerr J.F. (1992). Internucleosomal DNA cleavage should not be the sole criterion for identifying apoptosis. Int. J. Radiat. Biol..

[29] Crompton N.E., Ozsahin M. (1997). A versatile and rapid assay of radiosensitivity of peripheral blood leukocytes based on DNA and surface-marker assessment of cytotoxicity. Radiat. Res..

[30] Vermes I., Haanen C., Reutelingsperger C. (2000). Flow cytometry of apoptotic cell death. J. Immunol. Methods.

[31] Pengo P., Polizzi S., Battagliarin M., Pasquato L., Scrimin P. (2003). Synthesis, characterization and properties of water-soluble gold nanoparticles with tunable core size. J. Mater. Chem..

[32] Coulter J.A., Jain S., Butterworth K.T., Taggart L.E., Dickson G.R., McMahon S.J., Hyland W.B., Muir M.F., Trainor C., Hounsell A.R. (2012). Cell type-dependent uptake, localization, and cytotoxicity of 1.9 nm gold nanoparticles. Int. J. Nanomed..

[33] Lakshmanan I., Batra S.K. (2013). Protocol for apoptosis assay by flow cytometry using annexin V staining method. Bio-Protocol.

[34] Bevers E.M., Williamson P.L. (2016). Getting to the outer leaflet: Physiology of phosphatidylserine exposure at the plasma membrane. Physiol. Rev..

[35] Alshiraihi I., Kato T.A. (2022). Apoptosis detection assays. Chromosome Analysis: Methods and Protocols.

[36] Pan Y., Leifert A., Ruau D., Neuss S., Bornemann J., Schmid G., Brandau W., Simon U., Jahnen-Dechent W. (2009). Gold nanoparticles of diameter 1.4 nm trigger necrosis by oxidative stress and mitochondrial damage. Small.

